# Trait mindfulness and aggressive behavior in physical education undergraduates: a cross-sectional serial mediation analysis of anger rumination and self-control

**DOI:** 10.3389/fpsyg.2026.1823802

**Published:** 2026-06-01

**Authors:** Hanyang Cui, Wenting Liu, Luhui Li

**Affiliations:** 1 School of Physical Education, Shihezi University, Shihezi, Xinjiang, China; 2 School of Physical Education, College of Arts and Sciences Kunming, Kunming, Yunnan, China

**Keywords:** aggressive behavior, anger rumination, physical education undergraduates, self-control, trait mindfulness

## Abstract

**Background:**

Physical education undergraduates are frequently exposed to high-arousal, confrontational situations during training and competition, where aggressive behavior can disrupt athletic order and team functioning. Although trait mindfulness has been associated with aggression-related outcomes, the statistical pathways in sport contexts remain unclear. This study examined whether anger rumination and self-control showed statistical indirect effects in the association between trait mindfulness and aggressive behavior.

**Methods:**

A cross-sectional online survey was conducted among 786 physical education undergraduates at a university in Xinjiang, China, from February 13 to 20, 2026, via the Wenjuanxing platform. Trait mindfulness, anger rumination, self-control, and aggressive behavior were assessed, and confirmatory factor analysis supported the four-construct measurement model. A serial mediation model (mindfulness → anger rumination → self-control → aggressive behavior) was estimated using PROCESS Model 6 with 5,000 bootstrap resamples, controlling for sex and academic year.

**Results:**

Trait mindfulness was negatively associated with aggressive behavior (total effect, unstandardized *B* = −0.632, 95% CI [−0.689, −0.575]); it was also negatively associated with anger rumination and positively associated with self-control. The total indirect effect was significant (unstandardized *B* = −0.314, 95% CI [−0.362, −0.266]), as were indirect effects via anger rumination (*B* = −0.127, 95% CI [−0.165, −0.092]) and self-control (*B* = −0.136, 95% CI [−0.171, −0.101]) and the serial indirect effect (*B* = −0.051, 95% CI [−0.068, −0.037]). The direct association remained significant after accounting for the indirect effects (*B* = −0.318, 95% CI [−0.383, −0.252]).

**Conclusion:**

Higher trait mindfulness was associated with lower aggressive behavior, and this association was partly accounted for by lower anger rumination and higher self-control. Given the cross-sectional design, these findings should be interpreted as statistical associations and indirect effects rather than evidence of temporal ordering or causality; longitudinal and experimental studies are needed to test temporal ordering and causality.

## 1 Introduction

Violence and aggression during adolescence and young adulthood have long been regarded as priority concerns in global public health and social governance (World Health Organization, 2024). This risk is also pressing in campus and other youth-concentrated settings, where conflicts can escalate into substantial harm and legal consequences (Supreme People’s Court of China, 2016). In training and competition contexts, which are characterized by high arousal, direct confrontation, and frequent provocation cues, aggressive responses may be especially context-dependent. It is therefore necessary to situate aggression among physical education undergraduates within an interpretable psychological framework and to identify malleable psychological factors that may be associated with lower aggressive responding, thereby informing conflict prevention in sport settings and campus safety governance.

In provocative situations, anger rumination is associated with sustained hostility and arousal and lower self-control, and these tendencies are linked to aggressive responding (Denson et al., 2011). However, within the high-arousal and highly confrontational context of sport training and competition, it remains unclear whether higher trait mindfulness is associated with lower aggression, and whether anger rumination and self-control statistically account for this association within a single model. Several gaps remain particularly relevant to the present study. First, much of the existing evidence on mindfulness and aggression has been generated in general educational, occupational, clinical, or online contexts, whereas sport training and competition contexts have received less focused attention. Second, anger rumination and self-control have often been examined separately, leaving their combined and sequential roles insufficiently clarified. Third, evidence among physical education undergraduates remains limited, despite their frequent exposure to high-arousal and confrontational sport situations. By addressing these gaps, this study contributes to the sport psychology literature by testing a theory-informed serial indirect-effect model linking trait mindfulness, anger rumination, self-control, and aggression in this specific population. Guided by the process logic of the General Aggression Model, which emphasizes internal states, appraisal and decision processes, and behavioral outcomes, this study incorporated anger rumination as repetitive cognitive processing and self-control as an inhibitory gate within the same framework to characterize the statistical pathways linking trait mindfulness with aggression. Accordingly, the present study aimed to: (1) examine whether higher trait mindfulness is associated with lower aggression; (2) test the indirect effects of anger rumination and self-control (including their serial indirect effect) in the trait mindfulness–aggression association; and (3) provide evidence to inform process-oriented psychological skills training and conflict prevention tailored to sport training and competition contexts.

## 2 Literature review and research hypotheses

### 2.1 Mindfulness and aggressive behavior

Mindfulness is commonly defined as a psychological capacity to sustain awareness and attention to present-moment experience while adopting an accepting and nonjudgmental stance toward internal and external events. In sport training and competition contexts, where high arousal, direct confrontation, and recurrent conflict cues are more frequent, the association between mindfulness and aggression offers an important entry point for understanding sport-related conflicts and externalizing risk. Evidence across populations and methodological approaches has converged on a broadly consistent pattern. In school settings, mindfulness-based education has been linked to lower teacher-rated aggressiveness in children alongside improvements in attention and self-regulation (Suárez-García et al., 2020). In high-stress occupational groups such as law enforcement personnel, mindfulness-based resilience training has been associated with larger reductions in self-reported aggression than control conditions and has been accompanied by improvements in stress-related physiological and psychological indicators (Christopher et al., 2018). Randomized controlled evidence has further shown that mindfulness-based stress reduction is associated with decreases in aggressive anger expression (Robins et al., 2012). Beyond intervention studies, experimental and correlational findings are consistent with the same direction: trait mindfulness has been associated with lower aggressiveness and less hostile attribution bias, and a brief mindfulness induction in the context of social rejection has been followed by lower aggressive responding (Heppner et al., 2008). Longitudinal work has added developmental and contextual specificity, showing that positive parenting can be linked to lower subsequent aggression via higher mindfulness in adolescents (Su et al., 2022), and that mindfulness can buffer risk pathways in settings with readily activated provocation cues such as online interactions, such that the link between perceived discrimination and later cyberaggression is not evident at higher levels of mindfulness (Wang et al., 2022). Converging evidence from non-sport contexts also supports cross-context generality: higher trait mindfulness has been associated with less hostility and fewer counterproductive work behaviors, with hostile feelings acting as a relevant intervening process (Krishnakumar and Robinson, 2015). Theoretically, the General Aggression Model posits that person factors shape internal states and information processing, thereby influencing appraisal and decision processes; mindfulness, as a regulatory resource, may be associated with less hostile interpretation and lower reactive emotional intensity, supporting more adaptive behavioral choices. In parallel, reperceiving and decentering perspectives emphasize observing anger-related experiences and conflict cues without becoming absorbed in them, which may reduce the initiation of automatic response chains and constrain the behavioral expression of aggression. Based on this theoretical and empirical foundation, we proposed Hypothesis 1: Trait mindfulness is negatively associated with aggressive behavior.

### 2.2 Mediating role of anger rumination

Anger rumination refers to a dispositional tendency to repetitively replay anger episodes by focusing on provoking cues, revenge-related thoughts, and causal explanations. Such sustained processing can prolong anger arousal and increase the accessibility of hostile interpretations, creating a relatively stable cognitive “hot” state that is associated with aggressive responding. Reperceiving and decentering accounts highlight an observational stance toward emotional and cognitive content; accordingly, mindfulness may weaken prolonged recall and meaning-making that are pulled by anger, making individuals less likely to become trapped in anger-centered repetitive processing. Consistent with this view, evidence from a Japanese adult sample has shown that trait mindfulness is associated with lower anger rumination, and that anger rumination statistically accounts for the association between mindfulness and maladaptive anger reactions (Takebe et al., 2015), providing support for embedding anger rumination within a mindfulness-related pathway.

With respect to aggression, both associative network perspectives and the General Aggression Model suggest that rumination repeatedly activates anger–hostility–aggression linkages and increases the accessibility of aggressive scripts, which may bias appraisal and decision processes toward retaliatory choices. A multisystem integrative view further proposes that rumination is linked to aggression by sustaining hostile cognition, prolonging physiological arousal, and consuming self-regulatory resources (Denson, 2013). Experimental work manipulating post-provocation thought processes has shown that rumination, relative to distraction, increases triggered displaced aggression (Bushman et al., 2005), and that post-provocation rumination can elevate subsequent displaced aggression (Vasquez et al., 2013). Developmental and individual-difference findings also support the specificity of anger rumination to externalizing outcomes: anger rumination is more tightly linked to aggression than sadness-related rumination (Peled and Moretti, 2007) and shows stable associations with multiple forms of aggression (Anestis et al., 2009). In high-risk male samples, anger rumination has been closely tied to aggressive script rehearsal and aggressive behavior (Hosie et al., 2022). Longitudinal evidence further indicates that anger rumination can mediate the association between victimization/provocation-related experiences and later aggression (Quan et al., 2024). Taken together, prior work supports testing anger rumination as a potential explanatory process in the mindfulness–aggression association. Accordingly, we proposed Hypothesis 2: Anger rumination shows a significant indirect effect in the association between trait mindfulness and aggressive behavior.

### 2.3 Mediating role of self-control

Self-control refers to the capacity to inhibit impulses, delay responses, and maintain goal-directed behavior. In sport training and competition contexts characterized by frequent high arousal and confrontational cues, self-control may function as a critical regulatory “gate” that inhibits provocation-driven impulses from translating into behavioral output. Strength and resource models of self-control emphasize that self-regulation depends on finite executive resources and sustained attention; mindfulness, by enhancing present-moment awareness and attentional stability while reducing automatic impulsive responding and inefficient cognitive depletion, may support inhibitory control and executive functioning, and may be associated with greater inhibition of aggressive impulses during appraisal and decision processes in the General Aggression Model. Supporting evidence indicates that mindfulness meditation training yields overall gains in executive control in randomized controlled trial evidence, with relatively stronger improvements in inhibitory control (Cásedas et al., 2020). In high-risk samples of adolescent violent offenders, structured mindfulness training has also been associated with improved performance on inhibitory tasks such as stop-signal paradigms (Ron-Grajales et al., 2021).

More broadly, general self-control theory conceptualizes low self-control as a proximal risk factor for impulsivity and norm-violating behavior, with aggression as a prototypical externalizing manifestation that is more readily activated and expressed when self-control is insufficient. A meta-analysis has documented stable associations between self-control and delinquent/criminal behaviors, including aggression-related externalizing indicators, across both cross-sectional and longitudinal designs, providing quantitative support for self-control as a protective factor against broad externalizing risk (Vazsonyi et al., 2017). Experimental evidence likewise shows that when self-control resources are depleted and provocation is present, individuals are less able to inhibit aggressive impulses and display higher aggressive behavior, highlighting the proximal inhibitory function of self-control in the provocation-to-aggression transition (Barlett et al., 2016). Developmental evidence from three-wave longitudinal data further suggests that self-control predicts later reactive aggression and can be associated with cognitive–moral processes such as hostile rumination and moral disengagement (Cen et al., 2022). In addition, cross-sectional evidence among university students has shown that both mindfulness and self-control account for individual differences in aggressive tendencies (Kaya, 2022). Accordingly, we proposed Hypothesis 3: Self-control shows a significant indirect effect in the association between trait mindfulness and aggressive behavior.

### 2.4 Chain mediation effect of anger rumination and self-control

When explaining the mindfulness–aggression association, a single psychological component may not capture the full progression from emotional triggering to behavioral output; therefore, it is necessary to examine the sequential pathway involving anger rumination and self-control. Mindfulness emphasizes present-moment awareness and acceptance, and reperceiving/decentering, which involves approaching anger-related thoughts and conflict cues from an observer perspective, may reduce absorption in emotional content and lower the likelihood of rumination-based processing (Shapiro et al., 2006). When anger episodes are repeatedly rehearsed and elaborated, rumination may sustain hostility activation and consume limited self-regulatory resources, which may be linked to lower inhibitory control and goal-directed regulatory efficiency; in resource-based accounts of self-control, such sustained processing may constitute a depleting load and increase vulnerability to impulse-control failure (Baumeister et al., 2007). From an executive-function perspective, anger rumination has also been linked to deficits in task-switching and related control processes (Whitmer and Banich, 2007). In this way, anger rumination can be situated within the “internal state/cognitive processing” component of the General Aggression Model, whereas self-control maps onto the inhibitory gate within appraisal and decision-making, consistent with a serial pathway in which mindfulness is associated with lower anger rumination, which is associated with higher self-control, and in turn with lower aggression.

Empirical findings provide relevant evidence for this chain. Under provocation, rumination has been linked to increased aggressive responding and reduced self-control, and self-control has been reported to statistically account for the rumination–aggression association (Denson et al., 2011). At the trait level, the association between anger rumination and reactive aggression has also been partially explained by effortful control, suggesting that rumination and control-related capacities jointly relate to aggressive responding (White and Turner, 2014). Depletion of self-regulatory resources has been associated with reduced ability to inhibit aggressive impulses when insulted or provoked, highlighting the potential inhibitory role of self-control resources in provocative contexts (DeWall et al., 2007). A meta-analysis of Chinese student samples has further shown a stable, moderate negative association between self-control and aggression (Lei et al., 2020). In sport settings, where high arousal, confrontation, and provocation cues are denser and anger experiences are more likely to be repeatedly processed, athletes’ anger rumination and provocation experiences have both been associated with self-reported aggression (Maxwell, 2004). Although existing studies across paradigms and populations have supported key links such as mindfulness–rumination, rumination–control, and control–aggression, fewer studies have integrated mindfulness, anger rumination, and self-control within a single model to test their sequential pathway. Accordingly, we proposed Hypothesis 4: Anger rumination and self-control show a significant serial indirect effect in the association between trait mindfulness and aggressive behavior, following the specified pathway of trait mindfulness → anger rumination → self-control → aggressive behavior. This order was specified a priori based on theory, and alternative orders were not systematically compared in the current study.

**FIGURE 1:**
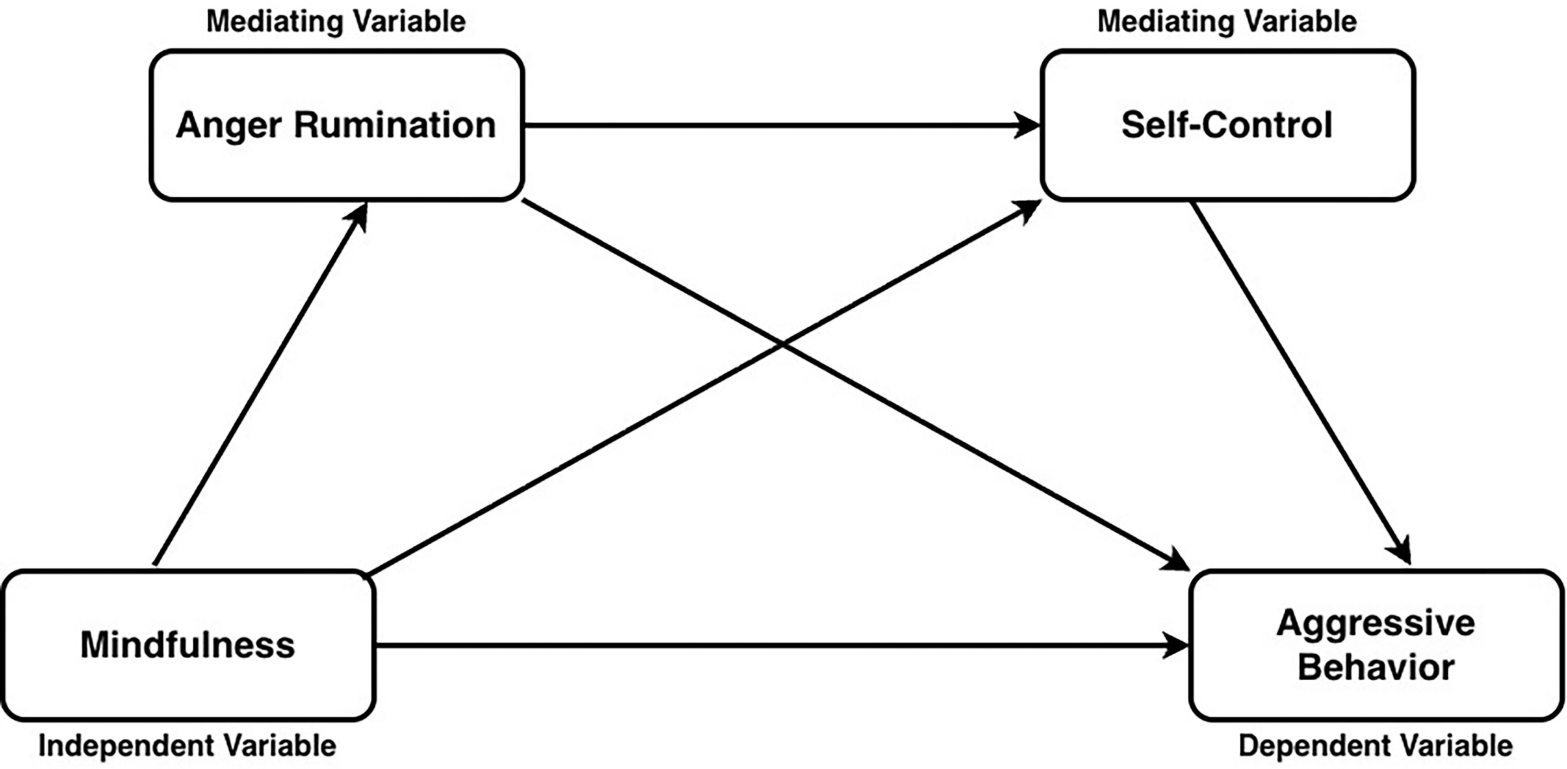
Hypothesized research model of the association between trait mindfulness and aggressive behavior through anger rumination and self-control.

**FIGURE 2:**
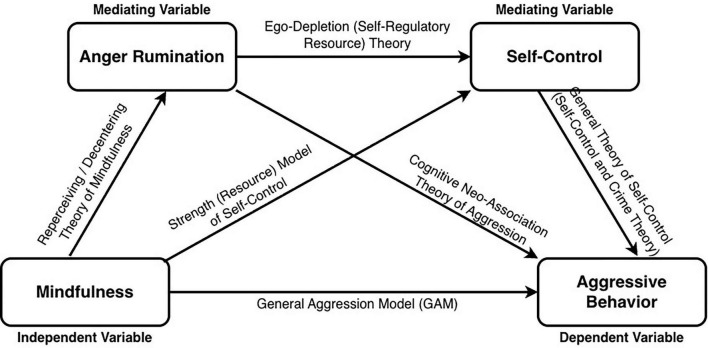
Theoretical rationale for the hypothesized serial mediation model linking trait mindfulness, anger rumination, self-control, and aggressive behavior.

Building on the evidence reviewed above, the mindfulness–aggression association has a replicable empirical basis, and anger rumination and self-control, which represent repetitive cognitive processing and inhibitory control, respectively, may jointly account for this association. Accordingly, we developed a conceptual framework (Figure 1 presents the hypothesized research model, and Figure 2 summarizes the theoretical rationale) and tested the following hypotheses in a sample of physical education undergraduates: (1) trait mindfulness is negatively associated with aggressive behavior; (2) anger rumination shows a significant indirect effect in the association between trait mindfulness and aggressive behavior; (3) self-control shows a significant indirect effect in the association between trait mindfulness and aggressive behavior; and (4) anger rumination and self-control show a significant serial indirect effect in the association between trait mindfulness and aggressive behavior, following the specified pathway of trait mindfulness → anger rumination → self-control → aggressive behavior.

## 3 Research subjects and methods

### 3.1 Research subjects

A questionnaire survey was conducted at Shihezi University, Xinjiang, China, from February 13 to 20, 2026. Participants were currently enrolled undergraduate students majoring in physical education at the School of Physical Education, Shihezi University, aged 18–25 years. Using cluster convenience sampling, recruitment was carried out through teaching classes or training units across grade levels. Standardized instructions were provided, and students completed an anonymous online questionnaire via the Wenjuanxing survey platform by scanning a QR code. Response time was automatically recorded by the platform and used for quality control. A total of 820 questionnaires were collected; 34 invalid questionnaires were excluded based on prespecified quality-control criteria, yielding 786 valid questionnaires (valid response rate = 95.85%). The final sample included 710 males and 76 females; 240 first-year, 235 second-year, 198 third-year, and 113 fourth-year students. Inclusion criteria were as follows: (1) currently enrolled as a physical education major at the School of Physical Education, Shihezi University and aged 18–25 years; (2) voluntary participation; and (3) complete responses to all questionnaire items. Exclusion criteria were as follows: (1) completion time < 5 min, which was considered insufficient for careful responding; (2) incomplete questionnaires or submissions with item-level missing data; (3) no informed consent or explicit refusal to participate; and (4) obvious logical inconsistencies or invalid response patterns. No questionnaire with item-level missing data was retained in the final analytic sample. The study protocol was reviewed and approved by the Science and Technology Ethics Committee of the First Affiliated Hospital of Shihezi University (Ethical Review No. KJ2026-062-01). Prior to data collection, all participants received a standardized explanation of the study purpose and procedures and were informed that participation was anonymous, fully voluntary, and confidential, and that the data would be used for research purposes only; participants could withdraw at any time without penalty. Informed consent was obtained electronically from all participants prior to questionnaire completion.

### 3.2 Measurement

#### 3.2.1 Mindfulness

Trait mindfulness was assessed using the Chinese 20-item short-form Five Facet Mindfulness Questionnaire (SF-FFMQ; Meng et al., 2020). The SF-FFMQ includes five facets: Observe, Describe, Act-with-Awareness, Non-Judging, and Non-Reactivity, with four items per facet. Items are rated on a 5-point Likert scale ranging from 1 (“never or almost never”) to 5 (“always or almost always”). Act-with-Awareness and Non-Judging were reverse-scored according to the scale instructions. After reverse scoring, the mean of the 20 items was computed as the overall mindfulness score, with higher scores indicating higher trait mindfulness. An example item is “I notice how foods and drinks affect my thoughts, bodily sensations, and emotions” (Observe). In the present sample, internal consistency was good for the five facets (Cronbach’s α = 0.878, 0.876, 0.874, 0.880, and 0.887, respectively), and confirmatory factor analysis showed acceptable fit (χ^2^/df = 2.331, RMSEA = 0.041, CFI = 0.977, TLI = 0.974).

#### 3.2.2 Anger rumination

Anger rumination was measured with the Anger Rumination Scale (ARS; Sukhodolsky et al., 2001), which has also been used in Chinese undergraduate samples (Wang et al., 2019). The ARS contains 19 items across four dimensions: Angry Memories, Thoughts of Revenge, Angry Afterthoughts, and Understanding of Causes. The original ARS uses a 4-point Likert format; to align response scales across instruments in the present study, the response format was adjusted to a 5-point Likert scale (1 = “strongly disagree,” 5 = “strongly agree”), following evidence that modest changes in the number of Likert response options have limited impact on psychometric properties (Preston and Colman, 2000). All items were scored in the same direction, and the mean of the 19 items was computed as the total anger rumination score, with higher scores indicating a stronger tendency toward anger-related repetitive thinking. An example item is “I ruminate about my past anger experiences” (Angry Memories). In the present sample, Cronbach’s α values for the four dimensions were 0.919, 0.864, 0.898, and 0.877, respectively, and CFA indicated good model fit (χ^2^/df = 1.492, RMSEA = 0.025, CFI = 0.992, TLI = 0.991).

#### 3.2.3 Self-control

Self-control was assessed using the Chinese version of the 13-item Brief Self-Control Scale (BSCS-13; Tangney et al., 2004; Unger et al., 2016). The BSCS-13 has a single-factor structure and uses a 5-point Likert scale (1 = “not at all like me,” 5 = “very much like me”). The scale includes both positively and negatively worded items; negatively worded items were reverse-scored according to the scoring guidelines. The mean of the 13 items was computed as the self-control score, with higher scores indicating higher self-control. An example item is “I am good at resisting temptation.” In the present sample, internal consistency was high (Cronbach’s α = 0.940), and CFA indicated good fit (χ^2^/df = 1.576, RMSEA = 0.027, CFI = 0.994, TLI = 0.992).

#### 3.2.4 Aggressive behavior

Aggressive behavior tendencies were measured using the Brief Aggression Questionnaire (BAQ; Webster et al., 2014; Webster et al., 2015; derived from Buss and Perry, 1992). The BAQ contains 12 items covering four dimensions: Physical Aggression, Verbal Aggression, Anger, and Hostility. The scale includes reverse-scored items, which were scored according to the BAQ scoring rules before computing scale scores. The original BAQ uses a 7-point Likert scale; to maintain consistency with other measures and reduce response burden, the response format was linearly compressed to a 5-point Likert scale (1 = “strongly disagree,” 5 = “strongly agree”), consistent with evidence that moderate compression of Likert response options has limited influence on reliability and validity (Preston and Colman, 2000). The mean of the 12 items was computed as the total aggression tendency score, with higher scores indicating higher aggression tendencies. An example item is “Given enough provocation, I may hit another person” (Physical Aggression). In the present sample, Cronbach’s α values for the four dimensions were 0.845, 0.854, 0.845, and 0.818, respectively, and CFA indicated good fit (χ^2^/df = 1.850, RMSEA = 0.033, CFI = 0.991, TLI = 0.988). Because the response formats of the ARS (4-point to 5-point) and BAQ (7-point to 5-point) were modified, absolute scores are not intended for direct comparison with studies using the original response options; the present study focuses on associations among variables within the current sample. Confirmatory factor analyses were used to evaluate the measurement structure, and subsequent regression/mediation analyses were conducted using observed composite scores (item means), consistent with a two-step approach.

### 3.3 Statistical analysis

All statistical analyses were performed in SPSS 26.0. Data were screened for quality, and descriptive statistics (means and standard deviations) were computed for mindfulness, anger rumination, self-control, and aggression. Because only complete questionnaires were retained in the final analytic sample, no missing-data imputation was performed. Harman’s single-factor test was applied to all items as a basic post hoc diagnostic of common method variance. Independent-samples *t*-tests were used to examine sex differences, and one-way analyses of variance were used to test differences across academic-year groups and age groups. Pearson two-tailed correlations were conducted to examine bivariate associations among the study variables. Multiple linear regression models were built with anger rumination, self-control, and aggression as dependent variables, respectively, and sex and academic year were included as covariates. The serial mediation model was estimated using the PROCESS macro (Model 6) with 5,000 bootstrap resamples, with mindfulness specified as the independent variable, aggression as the dependent variable, and anger rumination and self-control as mediators; indirect effects were evaluated using bootstrap 95% confidence intervals. Regression coefficients are reported as standardized β, whereas indirect effects are reported as unstandardized B with bootstrap confidence intervals. Internal consistency was assessed using Cronbach’s α, and construct validity was evaluated in AMOS 26 via confirmatory factor analysis, reporting χ^2^/df, RMSEA, CFI, and TLI. Statistical significance was set at *p* < 0.05 (two-tailed). Analyses were conducted at the individual level.

## 4 Results

### 4.1 Descriptive statistics for mindfulness, anger rumination, self-control, and aggressive behavior

Table 1 shows that, in the physical education undergraduate sample (*N* = 786; 710 males and 76 females), the overall mean scores (M ± SD) for mindfulness (Min), anger rumination (AR), self-control (SC), and aggressive behavior (AB) were 3.63 ± 0.85, 3.67 ± 0.86, 3.71 ± 1.03, and 3.68 ± 0.86, respectively. Independent-samples t tests indicated that females scored significantly higher than males on Min, AR, and SC (*t* = 2.933, *p* < 0.01; *t* = 2.150, *p* < 0.05; *t* = 2.141, *p* < 0.05), whereas the sex difference in AB was not significant (*t* = 0.414, *p* > 0.05). One-way analyses of variance further showed significant differences across academic-year groups for Min, AR, SC, and AB (*F* = 4.876, 5.230, 5.080, and 3.885, respectively; all *p* < 0.01), and significant differences across age groups for all four variables as well; the between-group difference was more pronounced for Min (*F* = 8.401, *p* < 0.001), and the differences for AR, SC, and AB were also significant (*F* = 4.429, 5.016, and 4.555, respectively; all *p* < 0.01).

**TABLE 1:** Descriptive statistics (M ± SD) and group comparisons for mindfulness (Min), anger rumination (AR), self-control (SC), and aggressive behavior (AB).

Group	N	Mindfulness (Min)	Anger rumination (AR)	Self-control (SC)	Aggressive behavior (AB)
Male	710	3.61 ± 0.85	3.65 ± 0.85	3.68 ± 1.04	3.67 ± 0.87
Female	76	3.90 ± 0.77	3.87 ± 0.87	3.95 ± 0.93	3.71 ± 0.76
Overall	786	3.63 ± 0.85	3.67 ± 0.86	3.71 ± 1.03	3.68 ± 0.86
Sex differences (t)		2.933**	2.150*	2.141*	0.414
Academic-year differences (F)		4.876**	5.230**	5.080**	3.885**
Age differences (F)		8.401***	4.429**	5.016**	4.555**

Values are presented as M ± SD. Sex differences were tested using independent-samples *t*-tests; academic-year and age-group differences were tested using one-way ANOVAs. *t*-values are reported as absolute values.

*p < 0.05, **p < 0.01, ***p < 0.001.

### 4.2 Common method bias test

Common method bias was evaluated using Harman’s single-factor test by entering all measurement items into an unrotated exploratory factor analysis (*N* = 786). The results yielded 14 factors with eigenvalues greater than 1, and the first factor accounted for 33.01% of the total variance, which was below the commonly used 40% heuristic threshold. This pattern did not suggest a dominant single-factor structure. Given that Harman’s single-factor test is a basic post hoc diagnostic, these results do not rule out common method variance but are reported as an initial assessment before the subsequent correlation analyses and regression/mediation analyses.

### 4.3 Correlation analysis of mindfulness, anger rumination, self-control, and aggressive behavior

As shown in Table 2, Min was significantly negatively correlated with AR (*r* = −0.459, *p* < 0.001), significantly positively correlated with SC (*r* = 0.639, *p* < 0.001), and significantly negatively correlated with AB (*r* = −0.597, *p* < 0.001). In addition, AR was significantly negatively correlated with SC (*r* = −0.559, *p* < 0.001) and significantly positively correlated with AB (*r* = 0.574, *p* < 0.001), whereas SC was significantly negatively correlated with AB (*r* = −0.618, *p* < 0.001). This correlational pattern was consistent with the hypothesized directions of the serial mediation model and provided a statistical basis for the subsequent regression equations and bootstrap tests of indirect effects.

**TABLE 2:** Means, standard deviations, and correlations among study variables.

Variable	M	SD	1	2	3	4
Mindfulness (Min)	3.63	0.85	1	1	1	1
Anger rumination (AR)	3.67	0.86	−0.459***			
Self-control (SC)	3.71	1.03	0.639***	−0.559***		
Aggressive behavior (AB)	3.68	0.86	−0.597***	0.574***	−0.618***	

*N* = 786. Pearson correlations (two-tailed) are reported. ***p < 0.001.

### 4.4 Testing the indirect effects of anger rumination and self-control in the association between mindfulness and aggressive behavior

**FIGURE 3:**
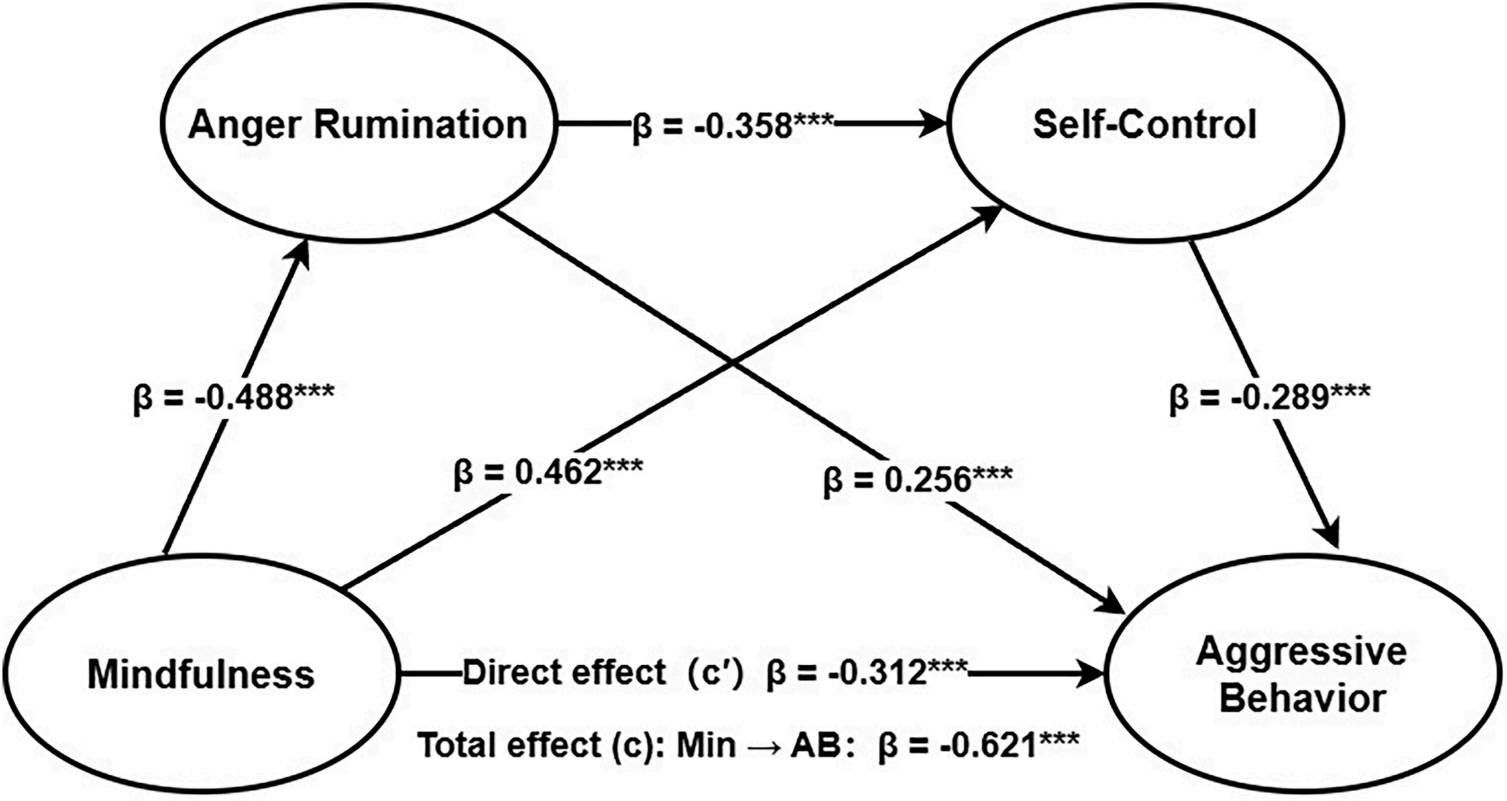
Regression results for the serial mediation model. Values on arrows are standardized regression coefficients (β) from PROCESS Model 6. Sex and academic year were included as covariates but are not shown. ***p < 0.001. *N* = 786. Indirect effects were tested using percentile bootstrap confidence intervals with 5,000 resamples.

After controlling for sex and academic year, the results first supported Hypothesis 1. Before the mediators were included, trait mindfulness (Min) was significantly and negatively associated with aggressive behavior (AB) [β = −0.621, *t*(782) = −21.828, *p* < 0.001], and the total-effect model was significant [*F*(3, 782) = 161.032, *R*^2^ = 0.382]. This finding indicated that higher trait mindfulness was associated with lower aggressive behavior among physical education undergraduates. We then estimated the serial mediation model in which the association between Min and AB was examined via anger rumination (AR) and self-control (SC) using PROCESS Model 6 with 5,000 bootstrap resamples in the physical education undergraduate sample (*N* = 786; Tables 3, 4 and Figure 3). In parallel, a confirmatory factor analysis of the four-construct measurement model was conducted in AMOS 26, showing good fit (χ^2^/df = 1.394, RMSEA = 0.022, CFI = 0.976, TLI = 0.975). Regression results are reported as standardized coefficients (β). In the equation with AR as the dependent variable, Min was negatively associated with AR [β = −0.488, *t*(782) = −15.517, *p* < 0.001], and the overall model was significant [*F*(3, 782) = 84.781, *R*^2^ = 0.245]. In the equation with SC as the dependent variable, Min was positively associated with SC [β = 0.462, *t*(781) = 15.870, *p* < 0.001], whereas AR was negatively associated with SC [β = −0.358, *t*(781) = −12.350, *p* < 0.001]; the model was significant [*F*(4, 781) = 199.892, *R*^2^ = 0.506]. In the equation with AB as the dependent variable, AR was positively associated with AB [β = 0.256, *t*(780) = 8.216, *p* < 0.001] and SC was negatively associated with AB [β = −0.289, *t*(780) = −8.197, *p* < 0.001]; after AR and SC were entered simultaneously, the direct association between Min and AB remained significant [β = −0.312, *t*(780) = −9.460, *p* < 0.001], and the overall model was significant [*F*(5, 780) = 169.525, *R*^2^ = 0.521].

**TABLE 3:** Regression results for the serial mediation model.

Variable	Anger rumination		Self-control		Aggressive behavior		Overall effect	
	β	*t*	β	*t*	β	*t*	β	*t*
Mindfulness	−0.488	−15.517***	0.462	15.870***	−0.312	−9.460***	−0.621	−21.828***
Anger rumination	–	–	−0.358	−12.350***	0.256	8.216***	–	–
Self-control	–	–	–	–	−0.289	−8.197***	–	–
*R* ^2^	0.245		0.506		0.521		0.382	
*F*	84.781***		199.892***		169.525***		161.032***	

Standardized regression coefficients (β) are reported. Sex and academic year were included as covariates in all models (not shown).

***p < 0.001.

**TABLE 4:** Indirect effects and effect decomposition in the serial mediation model.

Path	Unstandardized effect (B)	Proportion of total effect	95% CI	
			LL	UL
Min → AR → AB	−0.127	20.13%	−0.165	−0.092
Min → SC → AB	−0.136	21.50%	−0.171	−0.101
Min → AR → SC → AB	−0.051	8.12%	−0.068	−0.037
Total indirect	−0.314	49.75%	−0.362	−0.266
Direct effect (c’)	−0.318	–	−0.383	−0.252
Total effect (c)	−0.632	–	−0.689	−0.575

Effects are unstandardized (B). Indirect effects are reported with percentile bootstrap 95% confidence intervals based on 5,000 resamples. Proportions were computed as (indirect effect/total effect) × 100%. Sex and academic year were included as covariates. For reference, the completely standardized indirect effects (β_cs) were: total = −0.309 [−0.353, −0.264]; via AR = −0.125 [−0.161, −0.091]; via SC = −0.134 [−0.168, −0.099]; via AR→SC = −0.050 [−0.067, −0.036].

Bootstrap tests based on unstandardized coefficients (B) indicated that the total indirect effect was significant (*B* = −0.314, bootstrap 95% CI [−0.362, −0.266]), accounting for 49.75% of the total effect. All three specific indirect paths were significant: the indirect effect via AR (Min → AR → AB) was *B* = −0.127, 95% CI [−0.165, −0.092], accounting for 20.13%; the indirect effect via SC (Min → SC → AB) was *B* = −0.136, 95% CI [−0.171, −0.101], accounting for 21.50%; and the serial indirect effect (Min → AR → SC → AB) was *B* = −0.051, 95% CI [−0.068, −0.037], accounting for 8.12%. After including both mediators, the direct effect of Min on AB remained significant in unstandardized terms (c’ = −0.318, 95% CI [−0.383, −0.252]), whereas the total effect was c = −0.632, 95% CI [−0.689, −0.575], indicating that the Min–AB association comprised both indirect and direct components. Using the completely standardized indirect effect metric (β_cs), the total indirect effect was β_cs = −0.309, 95% CI [−0.353, −0.264], with β_cs = −0.125, 95% CI [−0.161, −0.091] via AR, β_cs = −0.134, 95% CI [−0.168, −0.099] via SC, and β_cs = −0.050, 95% CI [−0.067, −0.036] for the serial pathway. These results were consistent with H2, H3, and H4 in terms of the hypothesized directions; however, given the cross-sectional design, the indirect effects are interpreted as statistical associations rather than evidence of temporal ordering or causal processes.

## 5 Discussion

This study examined a process-informed statistical model of the mindfulness–aggression association in physical education undergraduates by integrating anger rumination and self-control within a single framework. Mindfulness (Min) reflects awareness of, and attention to, present-moment experience; anger rumination (AR) captures repetitive cognitive processing of anger-provoking events and cues; and self-control (SC) indexes regulatory capacity to inhibit impulses and sustain goal-directed behavior. The discussion is organized around the theory-informed sequence of mindfulness, rumination, self-control, and aggressive behavior (AB) to clarify the psychological meaning of each pathway in sport learning and training contexts. The findings indicated that the Min–AB association included both indirect and direct components: the total indirect effect accounted for 49.75% of the total effect, whereas the direct effect accounted for 50.25%. Among the three specific indirect pathways, Min → AR → AB contributed 20.13%, Min → SC → AB contributed 21.50%, and Min → AR → SC → AB contributed 8.12%, suggesting that the two parallel indirect paths accounted for most of the total indirect effect, whereas the serial path contributed a comparatively smaller proportion. Given the cross-sectional design, interpretations of these indirect effects are confined to statistical associations that are consistent with theory and do not establish temporal ordering or causal processes.

### 5.1 Mindfulness and aggressive behavior

In this sample of physical education undergraduates, mindfulness (Min) was significantly negatively correlated with aggressive behavior (AB) (*r* = −0.597, *p* < 0.001), and the total association between mindfulness and aggression remained significant after controlling for sex and academic year (β = −0.621, *p* < 0.001), indicating a robust pattern in which higher mindfulness was associated with lower aggressive tendencies. This pattern aligns with broader evidence: a meta-analysis reported a stable negative association between trait mindfulness and aggression and suggested that mindfulness-based interventions are associated with meaningful reductions in aggression (O’Dean et al., 2025). A systematic review likewise summarized an overall tendency for mindfulness or mindfulness-based interventions to relate to lower aggression while noting substantial heterogeneity across samples, measurement tools, and intervention protocols (Gillions et al., 2019). At the explanatory level, converging studies across populations suggest that mindfulness covaries with lower aggression and is often accompanied by less rumination, fewer emotion-regulation difficulties, or greater distress tolerance, which may help explain the statistical association between mindfulness and aggression (Kim et al., 2022; Garofalo et al., 2020; Brem et al., 2019). Evidence with clearer temporal ordering or contextual manipulation also supports this direction: experimental work in provocation paradigms observed that mindfulness training reduced behavioral aggression, and this change was not contingent on improved executive control (DeSteno et al., 2018); short-term longitudinal findings further indicated that a nonjudgmental orientation of mindfulness was associated with lower subsequent aggression perpetration (Hesse et al., 2021).

Within the General Aggression Model (GAM), mindfulness can be conceptualized as an individual resource that shapes internal states and information processing. By stabilizing attention and strengthening present-moment awareness, mindfulness may be associated with less hostile attribution and lower emotional reactivity, which may in turn reduce the accessibility of aggressive scripts and impulsive responding. From a decentering/reperceiving perspective, “observing without becoming entangled” may help individuals disengage from automatic affective and cognitive pulls when conflict cues arise, lowering the likelihood of entering an aggression response pathway. In high-arousal, highly confrontational sport training and competition settings, individuals are more frequently exposed to competitive stress and provocation cues; in student samples, higher physical activity frequency has also been reported to relate to higher aggressiveness indicators in bullying-related school settings (Méndez et al., 2019), which makes the emotion–cognition regulatory advantages implied by mindfulness particularly relevant. These points support the relevance of mindfulness-related skills for aggression prevention in sport training and competition contexts, consistent with applied evidence from high-stress occupations and high-risk groups (Ribeiro et al., 2020; Velotti et al., 2016).

Notably, although the present study identified a stable negative association between mindfulness and aggression, the direct association between mindfulness and aggression remained significant after anger rumination and self-control were included, suggesting that these two variables do not fully account for the overall association. Mindfulness may also relate to aggression through additional processes not modeled here, such as attentional selection to provocation cues, hostile interpretation bias, or thresholds for emotional reactivity. Thus, anger rumination and self-control should be viewed as partial explanatory processes rather than exhaustive mechanisms.

### 5.2 Indirect effect of anger rumination

Bootstrap tests indicated that anger rumination constituted a significant indirect pathway linking mindfulness and aggression (*B* = −0.127, 95% CI [−0.165, −0.092]), accounting for 20.13% of the total effect. This proportion was meaningful but still reflected only part of the overall association, and its substantive importance should therefore be interpreted cautiously. The direction of this pathway was consistent with the hypothesized model, providing additional support for H2. Conceptually, reperceiving/decentering emphasizes observing emotional and cognitive content without becoming immersed in it, which may reduce the tendency to enter rumination-like processing when anger cues arise. The cognitive neoassociation perspective further proposes that repeated activation of anger-related thoughts strengthens the anger–hostility–aggression associative network and increases the accessibility of aggressive cognitions, which may in turn be associated with aggressive responding; this account can be situated within the GAM sequence of “internal state–appraisal/decision–behavioral output” (Allen et al., 2018).

Empirically, multiple studies using student samples and diary-based follow-ups have reported that anger rumination statistically accounts for part of the association between mindfulness and aggression-related outcomes (Peters et al., 2015; Eisenlohr-Moul et al., 2016). Experience-sampling evidence further suggests that rumination not only co-occurs with momentary anger but also predicts subsequent anger responses, and that higher trait mindfulness attenuates the dynamic coupling between anger and rumination (Borders and Lu, 2017). In addition, the indirect pathway “mindfulness → lower anger rumination” has been shown to generalize to other outcomes such as forgiveness (de la Fuente-Anuncibay et al., 2021). With respect to how anger rumination may be linked to aggressive output, longitudinal findings indicate that anger rumination predicts subsequent overt harmful behaviors such as cyber aggression (Camacho et al., 2021); and in cognitive-processing chains, hostile attribution bias can further relate to reactive aggression via anger rumination (Quan et al., 2022). This pattern has also received cross-context support in online interaction and driving settings, where mindfulness has been linked to lower anger rumination, lower online trolling, and lower driving anger expression (Liu et al., 2022; Qu et al., 2024). Moreover, when self-regulatory resources are taxed, brief mindfulness induction has still been found to reduce behavioral aggression indices (Yusainy and Lawrence, 2015), and cross-sectional evidence likewise showed that dispositional mindfulness was negatively associated with aggressiveness among college students (Yu et al., 2022). The findings suggest that rumination identification, attention reorienting, and decentering/disengagement may be useful targets for reducing hostile script accessibility in sport-context interventions.

### 5.3 Indirect effect of self-control

Bootstrap tests further showed that self-control constituted a significant indirect pathway between mindfulness and aggression (*B* = −0.136, 95% CI [−0.171, −0.101]), accounting for 21.50% of the total effect. This proportion was slightly larger than that of the anger-rumination pathway but still indicated only partial explanatory scope. This pattern is consistent with the regression paths in which mindfulness was positively associated with self-control (β = 0.462, *t* = 15.870) and self-control was negatively associated with aggressive behavior (β = −0.289, *t* = −8.197), thereby supporting H3. The strength/resource account of self-control emphasizes that regulation depends on finite regulatory resources and efficiency in executive functioning; mindfulness may help maintain inhibitory control and behavioral restraint under provocation or high arousal by stabilizing attention, strengthening present-moment awareness, reducing impulse-driven automatic responding, and limiting inefficient emotion–cognition depletion. This account is consistent with higher-level evidence showing that mindfulness interventions yield reliable gains in executive domains such as sustained attention, working memory, and inhibitory control (Zainal and Newman, 2024), and with behavioral-task evidence linking trait mindfulness to better stop-signal inhibition performance (Logemann-Molnár et al., 2024). In studies that directly target aggression outcomes, classroom-based mindfulness training has been associated with reduced impulsivity and aggressive responses (Franco et al., 2016), and adolescent intervention research has suggested an indirect association between mindfulness and lower aggression via enhanced self-control (Zhang and Zhang, 2023). Evidence from Chinese samples also indicates that mindfulness may be indirectly associated with lower cyberbullying through self-control and that this pathway may vary by levels of cyberostracism (Xiong et al., 2023), collectively supporting the plausibility of a mindfulness–self-control–aggression pathway.

More broadly, general self-control theory treats low self-control as a proximal driver of impulsivity and norm-violating behavior; aggression, as a salient manifestation of externalizing problems, may be more readily expressed when self-control is insufficient. In naturalistic experience-sampling work, momentary decreases in self-control have been shown to predict a higher likelihood of aggressive ideation and verbal aggression (Plessen et al., 2023). Reviews of reactive aggression mechanisms similarly place inhibitory-control deficits and provocation-driven impulsive aggression within a common evidence chain (Bertsch et al., 2020). Under interpersonal stress, social exclusion can elevate aggression among college students by undermining self-control (Wang et al., 2025), and developmental evidence likewise suggests that family conflict is indirectly associated with adolescent aggression via reduced self-control (Sun et al., 2024). In the sport training and competition context of physical education undergraduates, the self-control pathway highlights inhibitory “gating” at high arousal, suggesting that mindfulness exercises, inhibitory-control training, and delayed-response strategies may help reduce the behavioral enactment of aggressive scripts.

### 5.4 Serial indirect effect of anger rumination and self-control

Bootstrap results showed a significant serial indirect effect of anger rumination and self-control in the mindfulness–aggression association (*B* = −0.051, bootstrap 95% CI [−0.068, −0.037]), accounting for 8.12% of the total effect. Although statistically significant, this proportion was comparatively modest, and its practical importance should therefore be interpreted cautiously. Because the model simultaneously included two parallel indirect pathways (Min → AR → AB and Min → SC → AB), the serial pathway required that both the AR → SC and SC → AB links hold for the indirect effect on AB to be observed; accordingly, its magnitude was jointly determined by multiple segments and appeared as a smaller component of the overall indirect-effect decomposition. This pattern was consistent with the regression directions in which anger rumination was negatively associated with self-control (β = −0.358) and self-control was negatively associated with aggression (β = −0.289) (both *p* < 0.001), providing additional support for H4. From a reperceiving/decentering perspective, observing emotional and cognitive content without becoming entangled may reduce the likelihood of entering rumination-like processing after anger cues emerge. At the same time, rumination has been linked to detectable associations with inhibition and switching components of executive functioning, suggesting a stable connection between repetitive processing and inhibitory-control processes (Vălenaş and Szentágotai-Tătar, 2017), such that lower self-control may be associated with the translation of aggressive impulses into behavioral output. Consistent with this view, the negative self-control–aggression association can be partially accounted for by “less rumination about anger-provoking events,” positioning rumination as a process node that links regulatory capacity and aggressive output (Li et al., 2019). Boundary evidence should also be acknowledged: although induced rumination can maintain anger experience, some work has not observed significant differences on specific behavioral inhibition tasks, suggesting that the rumination–self-control link may depend on measurement operationalization and task characteristics (Lievaart et al., 2017). At the outcome level, self-control has been repeatedly characterized as a protective factor against externalizing risks such as aggression in child and adolescent samples (Flores et al., 2020), and among athletes it has also been shown to explain individual differences in aggression and to function in an inhibitory manner (Sofia and Cruz, 2015).

In the training and competition context of physical education undergraduates, high arousal and confrontational cues increase the demand for sustained processing of anger experiences; when rumination persists, it may continuously occupy self-control resources, making it more likely that aggressive impulses shift from ideation to verbal or behavioral output. Cross-lagged evidence has reported that anger rumination predicts subsequent reactive aggression and that reactive aggression can in turn predict later angry rumination, potentially forming a reinforcing risk cycle (Wang et al., 2020). The pathway is also likely to vary across contexts: multi-method work suggests that mindfulness facets may sometimes operate primarily by weakening the “hostility → aggression” conversion and reducing maladaptive emotion-regulation strategies rather than solely through lowering rumination (Liang et al., 2018), which is consistent with the present pattern in which the serial pathway was significant yet accounted for a relatively modest proportion of the total effect. These findings also suggest that anger rumination may be considered a proximal intervention target in sport settings (Fresnics and Borders, 2017). Overall, the serial pathway “mindfulness → anger rumination → self-control → aggression” can be understood as a theory-consistent statistical pathway linking anger-related repetitive processing and the gating role of self-control. Compared with models focusing on a single mediator, this serial model provides a more differentiated framework and more specific intervention targets for identifying and managing aggression risk in high-arousal, highly confrontational sport training and competition settings.

## 6 Practical implications

The present study contributes to understanding the psychological correlates and theory-consistent indirect pathways associated with aggression among physical education undergraduates, and it offers practice-oriented implications for university-level talent development in physical education, routine team management, and campus conflict prevention. The findings are consistent with a pathway involving mindfulness, anger rumination, self-control, and aggressive behavior, linking repetitive cognitive processing of anger-related events with the inhibitory role of self-control within a single framework. By linking cognitive–emotional processing to behavioral choice, this sequence adds process-specific clues to accounts grounded in the General Aggression Model. From an applied perspective, the study suggests that mindfulness-based practice may be considered alongside rumination management and self-control strengthening in training and competition settings. Given the cross-sectional, self-report design, the recommendations below are derived from theory-consistent associations and should be implemented alongside team-based contextual assessment and ongoing monitoring.

For physical education undergraduates and sport team training practice, practice initiatives may be organized around key steps of awareness, disengagement, and delayed responding and embedded into routine training workflows: (1) integrate brief mindfulness practices into warm-ups, breaks between drills, and pre-competition preparation to establish a fixed routine of attentional resetting and breath awareness, with the aim of reducing impulse-driven responding under high arousal; (2) develop contextualized training modules targeting anger rumination, using scenarios such as disputed officiating, confrontational incidents, and post-error reviews to rehearse operational steps such as identifying rumination cues, labeling emotions and thoughts, and returning attention to the present task, which may help limit sustained activation of hostile scripts; (3) strengthen behavioral strategies for self-control at conflict points by using pause cues, delayed-response rules, and implementation intentions, such as taking three breaths before responding when provocation cues are noticed, so that inhibitory control is translated into actionable behavioral norms; and (4) incorporate nonjudgmental reflection and goal-oriented planning into post-training debriefs, distinguishing factual review from rumination loops and helping athletes shift attention from retaliatory processing to improvable skills and strategies.

At the institutional level, a coordinated support system that integrates curriculum, training, management, and services may also be considered: incorporate mindfulness, emotion regulation, and self-control into sport psychology coursework and freshman orientation, and provide coaches with targeted training in conflict communication and emotion management so that training settings adopt a consistent set of norms and shared language. In team management, early identification and stepped-care support mechanisms may be established, offering group-based programs and individual counseling for students with frequent conflicts, pronounced rumination tendencies, or weaker self-control, and program effects may be evaluated using multi-source indicators such as peer and coach ratings and disciplinary incident records. At the campus governance level, institutions may strengthen safe sport policies by synchronizing anti-aggression norms, sportsmanship and discipline education, and accessible psychological support channels to support order and developmental quality in training and competition environments.

## 7 Limitations and future directions

This study examined the association between trait mindfulness and aggressive behavior in a sample of physical education undergraduates and further identified significant indirect effects involving anger rumination and self-control. Although these findings are consistent with a process-oriented account of aggression-related outcomes, five limitations should be considered. First, all core variables were assessed primarily through self-report questionnaires. Second, the cross-sectional design precludes conclusions about temporal ordering or causality. Third, covariate adjustment was limited, and possible class- or training-unit-level clustering was not explicitly modeled. Fourth, the theory-driven serial model was not systematically compared with alternative model specifications. Fifth, the sample was drawn from one university in one region of China and had an imbalanced sex distribution, which may limit generalizability. These issues provide directions for future research using more rigorous, multi-source, and context-sensitive designs.

### 7.1 Limitations of measurement methods

Measures of mindfulness, anger rumination, self-control, and aggressive behavior relied primarily on self-report questionnaires administered at a single time point. This approach emphasizes subjective experience and self-evaluation and may not adequately capture behavioral indices of inhibitory control or context-triggered aggressive responses. In addition, aggression-related items are susceptible to social desirability and impression management, which may lead to systematic underreporting. Generic measures may also be less sensitive to the specific manifestations of aggression in high-arousal, highly confrontational training and competition settings. Moreover, the outcome measure focused on general aggression tendencies and did not distinguish rule-compliant physical contact inherent in competitive sport from rule-violating aggression or broader externalizing problem behaviors. Because the response formats of the ARS and BAQ were modified, absolute scores are not directly comparable with studies using the original response options, although the present study provided internal consistency and CFA evidence for the modified measures. In addition, the common method bias diagnostic used in this study was limited and cannot fully rule out common method variance. Future studies should strengthen measurement validity by incorporating coach/peer ratings, disciplinary or incident records, experience sampling, behavioral inhibition tasks, physiological indicators, and more robust common-method controls such as marker variables, latent method factors, or multi-source designs.

### 7.2 Limitations in study design and causal inference

A cross-sectional survey design was used, and mindfulness, anger rumination, self-control, and aggressive behavior were assessed concurrently. Accordingly, the serial mediation model describes statistical associations and indirect effects consistent with the proposed framework rather than temporal ordering or causal processes, and it cannot rule out reverse effects or shared variance driven by unmeasured third variables. Therefore, the regression-based model should be interpreted as an indirect-effect model rather than as evidence of causal mediation; future studies should test temporal precedence and pathway direction using multi-wave longitudinal designs or context-based experimental manipulations.

### 7.3 Limited adjustment for confounding factors and potential non-independence

Although sex and academic year were controlled in model testing, aggressive behavior among physical education undergraduates is likely shaped by a broader set of individual and contextual factors, and the current covariate adjustment remains limited. In addition, because participants were recruited through classes or training units, observations may not have been fully independent; clustering was not explicitly modeled in the present analyses, and standard errors may therefore have been underestimated, potentially inflating statistical significance. Important unmeasured factors may include trait anger, impulsivity, hostile attribution bias, personality traits, prior conflict exposure, injury status, sport type and contact level, competitive level, training load, coaching style, team norms, and peer conflict exposure. Future studies should measure these covariates and consider stratified analyses, moderation analyses, or multilevel analyses to account for individual- and class/team-level influences.

### 7.4 Model robustness and hypothesis testing

The study tested a theory-driven serial pathway (mindfulness → anger rumination → self-control → aggressive behavior), but model robustness should be examined further. Although the hypothesized order was specified a priori, current inferences relied mainly on regression-based estimates and bootstrap confidence intervals, and parallel, reverse, or statistically equivalent alternatives were not systematically compared. Sensitivity analyses under alternative exclusion criteria and invariance analyses across subgroups, such as sex, academic year, or sport type, were also not conducted. In addition, AMOS was used to evaluate the four-construct measurement model, whereas structural paths and competing models were not tested within a full structural equation modeling framework. Future research should compare competing models, conduct invariance and sensitivity analyses, and use cross-validation or replication samples to strengthen the robustness and reproducibility of hypothesis tests.

### 7.5 Sample and generalizability

Participants were drawn from physical education undergraduates at a single university in Xinjiang, China. The sampling frame and cultural context were therefore relatively concentrated, and the sex distribution was imbalanced, which may limit generalization to universities in other regions, non–physical education undergraduates, or athlete populations with different competitive levels. Differences across institutions and regions in sport type, training intensity, and team management practices may also shape how mindfulness, anger rumination, self-control, and aggression relate to one another. Future studies should adopt multi-site and multi-region sampling, increase the representation of women and diverse sport disciplines, and compare physical education students with other student or athlete populations.

### 7.6 Future research directions and applications

Building on these limitations, future research should prioritize four directions. First, longitudinal, experimental, or randomized intervention designs are needed to test temporal ordering and pathway direction. Second, sport-context-specific aggression outcomes should be refined by distinguishing reactive versus proactive aggression, verbal versus physical aggression, and competition-related versus daily-life aggression. Third, boundary conditions such as sport contact level, competitive level, training load, coaching style, and team norms should be examined using moderation models or, where appropriate, multilevel models. Fourth, comparative intervention studies should evaluate mindfulness training, rumination-interruption training, and inhibitory-control training separately and in combination, using objective indicators such as disciplinary incidents, conflict records, and behavioral task performance.

## 8 Conclusion

This study examined the association between trait mindfulness and aggressive behavior among physical education undergraduates and incorporated anger rumination and self-control within a single model to examine potential indirect pathways from the perspectives of repetitive cognitive processing and behavioral inhibition. The findings indicated that higher trait mindfulness was associated with a lower tendency toward aggressive behavior. Anger rumination and self-control each showed significant indirect effects, and a significant serial indirect effect was also observed, such that lower anger rumination was associated with greater self-control, which in turn was associated with lower aggressive behavior. These findings are consistent with the process logic of the General Aggression Model and suggest potential targets for sport psychological skills training and conflict prevention in athletic teams.

Future research should employ longitudinal tracking and randomized controlled or context-based provocation designs, integrate multi-informant ratings and behavioral indicators, and test and refine the proposed pathway and its boundary conditions in broader sport populations and more diverse training and competition settings.

## Data Availability

The original contributions presented in this study are included in the article; further inquiries can be directed to the corresponding author.
